# Effect of different levels of EDTA on phytoextraction of heavy metal and growth of *Brassica juncea* L.

**DOI:** 10.3389/fmicb.2023.1228117

**Published:** 2023-08-03

**Authors:** Mohab Amin Kamal, Kahkashan Perveen, Faheema Khan, R. Z. Sayyed, Ong Ghim Hock, Santosh Chandra Bhatt, Jyoti Singh, Mohd Obaid Qamar

**Affiliations:** ^1^Department of Civil Engineering, College of Engineering, King Saud University, Riyadh, Saudi Arabia; ^2^Department of Botany and Microbiology, College of Science, King Saud University, Riyadh, Saudi Arabia; ^3^Faculty of Health and Life Sciences, INTI International University, Nilai, Negeri Sembilan, Malaysia; ^4^Faculty of Agriculture, Kumaun University, Nainital, Uttarakhand, India; ^5^Department of Microbiology, College of Basic Sciences and Humanities, G. B. Pant University of Agriculture and Technology, Pantnagar, India; ^6^Department of Civil Engineering (Environmental Science and Engineering), Yeungnam University, Gyeongsan, Republic of Korea

**Keywords:** heavy metals, EDTA, oxidative stress, phytoremediation, *Brassica juncea*, SDG

## Abstract

Heavy metal pollution of soil is a major concern due to its non-biodegradable nature, bioaccumulation, and persistence in the environment. To explore the probable function of EDTA in ameliorating heavy metal toxicity and achieve the sustainable development goal (SDG), *Brassica juncea* L. seedlings were treated with different concentrations of EDTA (0, 1.0, 2.0, 3.0, and 4.0 mM Kg^−1^) in heavy metal-polluted soil. Plant samples were collected 60 days after sowing; photosynthetic pigments, H_2_O_2_, monoaldehyde (MDA), antioxidant enzymes, and ascorbic acid content, as well as plant biomass, were estimated in plants. Soil and plant samples were also examined for the concentrations of Cd, Cr, Pb, and Hg. Moreover, values of the phytoremediation factor were utilized to assess the accumulation capacity of heavy metals by *B. juncea* under EDTA treatments. In the absence of EDTA, *B. juncea* seedlings accrued heavy metals in their roots and shoots in a concentration-dependent manner. However, the highest biomass of plants (roots and shoots) was recorded with the application of 2 mM kg^−1^ EDTA. Moreover, high levels (above 3 mM kg^−1^) of EDTA concentration have reduced the biomass of plants (roots and shoots), photosynthetic area, and chlorophyll content. The effect of EDTA levels on photosynthetic pigments (chlorophyll a and b) revealed that with an increment in EDTA concentration, accumulation of heavy metals was also increased in the plant, subsequently decreasing the chlorophyll a and b concentration in the plant. TLF was found to be in the order Pb> Hg> Zn> and >Ni, while TF was found to be in the order Hg>Zn>Ni>Pb, and the best dose was 3 mM kg^−1^ EDTA for Hg and 4 mM kg^−1^ for Pb, Ni, and Zn. Furthermore, hyperaccumulation of heavy metals enhanced the generation of hydrogen peroxide (H_2_O_2_), superoxide anions (O_2_^•−^), and lipid peroxidation. It also interrupts mechanisms of the antioxidant defense system. Furthermore, heavy metal stress reduced plant growth, biomass, and chlorophyll (chl) content. These findings suggest that the exogenous addition of EDTA to the heavy metal-treated seedlings increases the bioavailability of heavy metals for phytoextraction and decreases heavy metal-induced oxidative injuries by restricting heavy metal uptake and components of their antioxidant defense systems.

## 1. Introduction

In the recent past, rapid industrialization and enhanced urbanization have given rise to an amplified level of heavy metal (HM) contamination in the ecosystem, which has arisen as a global concern (Saleem et al., 2018). HMs contamination of soil is a key concern due to their nonbiodegradable nature, bioaccumulation, and perseverance in the ecosystem (Din et al., 2020). HMs contamination has augmented in the soil as well as in the water because of the release of HMs encompassing effluents from several industries, namely alloying, electroplating, metallurgy, paints, tanneries, textile dyes, and timber processing (Ganesh et al., 2009; Gill et al., 2016). Various toxic HMs existing in diverse oxidation states, such as arsenic (As), cadmium (Cd), chromium (Cr), copper (Cu), mercury (Hg), nickel (Ni), lead (Pb), and zinc (Zn); radioactive elements, namely uranium and strontium; along with organic compounds like trinitrotoluene, 1,3,5-trinitro-1,3,5-hexahydrotriazine; petroleum hydrocarbons (benzene, toluene, xylene, etc.), all are hard to eliminate from the ecosystem due to their nonbiodegradable nature, and they become acutely toxic if their concentration exceeds a certain threshold. Due to their hydrophilic nature and prevalent mobility, HMs can simply enter the rhizospheric region of plants, be transported to the shoot part of plants, and become a serious risk for living organisms, including humans, by food-chain transfer (Singh and Singh, 2017). Human exposure to HMs comes frequently via different food crops, which accounts for approximately 89% of the total intake, whereas the remaining 11% arises via skin contact and breathing of polluted dust. These HMs may commonly react with biological systems via losing one or more electrons and forming metal cations which have affinity to the nucleophilic sites of vital macromolecules. Several acute and chronic toxic effects of heavy metals affect different body organs. Birth defects, cancer, gastrointestinal, immune system and kidney dysfunction, nervous system disorders, skin lesions, and vascular damage are examples of heavy metals toxic effects (Balali-Mood et al., 2021; Mitra et al., 2022). Various studies have proved that surplus quantities of HMs contamination negatively influence the physiology and phenotype of some plants. The general phenotypic signs of HM-induced stress are chlorosis, epinasty of leaves, disturbance in tube growth along with pollen germination, necrosis, and stunted plant growth. HMs reduces nutrient uptake, disturbs chlorophyll (chl) content, abolishes the ultrastructural mechanisms of the chloroplast, and changes nitrogen and sulfur metabolism, thereby damaging the photosynthetic process and hindering the metabolism of plants (Benavides et al., 2005; Singh et al., 2022). As few heavy metals are redox-inactive metals, they can indirectly produce more reactive oxygen species (ROS), which contain singlet oxygen (^1^O_2_), hydrogen peroxide (H_2_O_2_), superoxide anion (O_2_^•−^), and the hydroxyl radical (OH^•^).

The phytoremediation method utilizes plants (with or without the associated microorganism) for the removal of notorious contaminants. It has been extensively accepted and applied in the last few years. As this methodology is economical, sustainable, eco-friendly, and unintrusive, it is anticipated to play a vital role concerning industrial scale if executed with proper consideration (type of pollutant, composition of waste generated, seasonal variation, and diversity of plants to be utilized) (Lin et al., 2002; Yang et al., 2017). This method eradicates HMs by taking benefit of several plants ability to absorb and accrue metals and congregating them within the plant biomass. The perspective of this type of remediation technique is to decrease the concentration of HMs from polluted soil so that these plants can be utilized favorably for agriculture, horticulture, forestry, grazing, etc. Several plant species are capable of developing several approaches to combat HMs toxicity and reduce hostile effects by evading toxicity via metal-binding on the cell walls, averting transport across cell membranes, active efflux, compartmentalization and excretion methods as well as by internal metal chelation (Singh and Singh, 2017).

At present, several researchers are focused on the supportive treatments for phytoremediation by utilizing genetic engineering, sorbents, phytohormones, microbiota, microalgae or nanoparticles. In future, purification of soils on an industrial scale will most likely be possible through genetically modified organisms. However, there is a substantial risk of gene transfer from transgenic plants or microorganisms to the environment. Engineering bioremediation offers few operative solutions in the form of the use of various organic substances (e.g., sewage sludge, sorbents, enzymatic and microbial preparations or nanoparticles). Moreover, few research related to new techniques such as *in situ* solar driven technology make use of vascular plants to accumulate and translocate metals from root to shoot. Harvesting the plant shoots can permanently remove these contaminants from environment (Jadia and Fulekar, 2009; Mocek-Płociniak et al., 2023).

Plants have a coordinated and multifaceted antioxidant defense system to sustain reactive oxygen species (ROS) at a steady-state level by rummaging the generated ROS to cope with oxidative stress (Gill and Tuteja, 2010; Ma et al., 2017; Ashraf et al., 2021). The defense system, containing enzymes namely ascorbate peroxidase (APOX), catalase (CAT), dehydroascorbate reductase (DHAR), glutathione peroxidase (GPOX), glutathione reductase (GR), monodehydroascorbate reductase (MDHAR), guaicol peroxidase (POD) and superoxide dismutase (SOD) has an imperious part in rummaging the produced ROS because of metal toxicity (Gill and Tuteja, 2010). Numerous studies have reported different functions of several antioxidant enzymes in diverse species of plants under HM stress conditions (Gill et al., 2015; Kanwar et al., 2015; Handa et al., 2018). Non-enzymatic antioxidants such as ascorbic acid and glutathione tocopherols also function in a harmonized way with various enzymatic antioxidants to counterbalance HM-induced ROS. For example, ascorbic acid and glutathione levels were enhanced under heavy metal stress conditions (Ashger et al., 2018; Ulhassan et al., 2019). These antioxidant enzymes diminish oxidative damage encouraged by ROS. Cellular macromolecules, *viz.* proteins, lipids, and nucleic acids, are oxidized through the elevated level of ROS (Sigfridsson et al., 2004; Hasanuzzaman et al., 2012). These enzymes act as quenchers of lipid and ROS radicals (Noctor and Foyer, 1998; Smirnoff, 2000; Hollander-Czytko et al., 2005). *B. juncea* is a fast-growing crop with medicinal and oil-yielding properties. It produces a high amount of biomass and has a robust and well-studied antioxidant defense system. However, heavy metal contamination results in a noteworthy loss of yield (Bhuiyan et al., 2011; Shekhawat et al., 2012).

Hyperaccumulation of these heavy metals can be encouraged with the addition of chemical alteration, such as ethylene diamine tetraacetic acid (EDTA), as a plant substrate to formulate a soluble or insoluble target metal, *viz.* Pb (Christopher et al., 1998). EDTA generally act as chemical chelator for eradicating toxic HMs from contaminated soil systems, predominantly where there is less metal bioavailability (Hernandez-Allica et al., 2007). EDTA function in the uptake of HMs and reducing its toxicity has already been documented in some plants (Dipu et al., 2012; Shahid et al., 2014; Khan et al., 2019). Moreover, different metals’ solubility is augmented by EDTA in the soil system, which enhances their uptake, bioavailability, and translocation from the rhizospheric region to the shoots in most vascular plants (Farid et al., 2015). EDTA has been utilized in plentiful experiments with diverse species of the Brassicaceae family as a metal chelator for monitoring a range of biochemical and physiological parameters. Contrariwise, Evangelou et al. (2007) stated that Cd accumulation is inhibited by EDTA in *Nicotiana tabacum* L. plants. Correspondingly, this chelating agent was also detected to alter Cd uptake by conquering Cd toxicity in several plant species, namely *Beta vulgaris* L., *Oryza sativa* L., *Phaseolus vulgaris* L., and *Vigna unguiculata* (L.). Moreover, several studies have reported that EDTA improves the antioxidant defense mechanism and growth of plants under HMs stress conditions (Hardiman and Jacoby, 1984; Greger and Lindberg, 1986; Weihong et al., 2009; Xu et al., 2010; Agbadah et al., 2016). Therefore, the aim of this study was to investigate the possible role of EDTA in mitigating HMs toxicity and its effect plant health. In view of this, we examined the effect of different EDTA levels on metal accumulation, physiological parameters, and the antioxidant defense mechanism of *B. juncea* in heavy metal contaminated soil.

## 2. Materials and methods

### 2.1. Plant material, physicochemical analysis, and experimental conditions

The collection of soil was done from a commercial horticulture field in Cairo, Egypt. Soil was oven-dried at 35°C for 4 days and filtered through a 6 mm mesh. The physico-chemical properties of soil were assayed as per the method described by Merkl et al. (2005). The soil had the properties of pH (7.4), Electrical conductivity (1.13 dsm^−1^), total nitrogen (0.09%), total phosphorus (0.78%), organic carbon 0.487% and Zn 2.7, Fe 708.45, Mn 44.7, Ni 0.5 mg kg^−1^ dw, respectively. Afterwards, 5 different gradients of EDTA (0, 1, 2, 3, and 4 mM kg^−1^) were prepared by adding the respective weight of the EDTA salt (292.2 g EDTA to 1 kg of water to prepare 1 M kg^−1^) to distilled water to prepare the different gradients and applied to the soil. Moreover, wastewater from the industry was collected in a propylene container from El Tebin industrial area, Egypt. The quantity of heavy metals present in the chemical wastewater was analyzed by inductive coupled plasma mass spectrometer (ICPMS). The composition of heavy metals were 12.36 Pb, 10.64 Ni, 30.45 Zn and 6.5 Hg mgL^−1^ and their physicochemical parameters were pH 6.2 and chemical oxygen demand (COD) 1917, biological oxygen demand (BOD) 196, Cl 1895, Ca 824.5 and Mg 586.2 mgL^−1^. The tap water had the properties of pH 6.9, total dissolved solids 145, total hardness 240, calcium hardness 106, dissolved oxygen 3.6, chloride ion 83, alkalinity 110, Na 25 and K 6 mgL^−1^.

Seeds of *B. juncea,* cultivar Balady were obtained from the commercial market, in Cairo, Egypt for conducting *in situ* pot experiments. 5 seedlings were maintained in each pot and the experiment was executed using a completely randomized design (CRD) with three replications. Seeds of *B. juncea* were sown in pots sized 15 × 15 cm, with 15 kg of garden soil in each pot and different amendments of heavy metals. Irrigation of each pot was done with a constant quantity of 5 L of industrial wastewater per day at the same time for 90 days. The implications of water as well as industrial wastewater levels were constant across all treatment pots.

#### 2.1.1. Plant sampling

Plant samples were collected after 60 DAS (days after sowing) from each pot and washed repeatedly using tap water to eliminate unwanted debris for the estimation of photosynthetic pigments. Soil and plant samples were again collected at 90 DAS to examine heavy metal concentrations of Cd, Cr, Pb, and Hg, as well as plant biomass.

### 2.2. Measurement of heavy metal content transfer factor and translocation factor

For heavy metal analysis in plants, 1 g of plant samples was dried out and finely grinded in an electric grinder, then digested in HNO_3_:HClO_4_ (3:1, v/v) at 80°C. Metal concentrations (Pb, Ni, Zn, and Hg) in plant samples were analyzed by means of an Inductively-Coupled Plasma Mass Spectrometer, Perkin Elmer Corporation (ICP Optima 3,300 RL). For metals, a standard reference material (E-Merck, Germany) was utilized for calibration and quality assurance for each analytical investigation. The detection limits of Hg, Pb, Ni, Zn, and Zn were 0.01, 0.1, 0.5, and 2.0 μg/L, respectively. Replication analysis (*n* = 5) was done to measure the precision of the analytical techniques. Triplicate analysis for each metal varied by not more than 5%. The treatments of EDTA were adjusted to different values in contrast with the controls and repeated three times (Al Mahmud et al., 2019).


$$\mathrm{TF}=\left[\begin{array}{l} \left(\mathrm{Metal concentration}\;\left(\mathrm{root}+\mathrm{shoot}\right)\mathrm{,mg}\;{\mathrm{kg}}^{-1}\right)/\\ \left(\mathrm{Metal concentration of soil,mg}\;{\mathrm{kg}}^{-1}\right) \end{array}\right]$$

$$\mathrm{TLF}=\left[\begin{array}{l} \left(\mathrm{Metal concentration in the shoots,mg}\;{\mathrm{kg}}^{-1}\right)/\\ \left(\mathrm{Metal concentration in the roots,mg}\;{\mathrm{kg}}^{-1}\right) \end{array}\right]$$

### 2.3. Estimation of photosynthetic pigments content

Leaf chlorophyll content was estimated as per the procedure described by Hiscox and Israelstam (1979). 0.05 g of leaf tissue from each treatment was weighed and chopped in a test tube containing 10 mL of dimethyl sulfoxide (DMSO) and incubated for 3 h at 60°C. The absorbance was analyzed through a spectrophotometer at 645 and 663 nm. The chlorophyll content was calculated using the following formula:


Chlorophyll a=12.7xA663−2.69xA645xV1000xW

Chlorophyll b=22.9xA645−4.68xA663xV1000xW

Where:

A = Absorbance at specific wave length,

V = Final volume of solution,

W = fresh weight of tissue.

### 2.4. Estimation of H_2_O_2_ and monoaldehyde (MDA) content

Estimation of H_2_O_2_ content was done through the method given by Velikova et al. (2000). The fresh leaf tissues were homogenized in 1.5 ml of tri-chloroacetic acid (0.1%) and centrifuged at 4°C for 15 min at 12,000 rpm. 0.4 ml of supernatant was supplemented with equal volume of 10 mM PPB (Potassium Phosphate Buffer) and 0.8 mL of potassium iodide (KI, 1 M). Absorbance of reaction mixture was observed at 390 nm. Concentration of H_2_O_2_ was examined by preparing standard curve of H_2_O_2_.

The MDA level was examined via Heath and Packer (1968) method. One gram of fresh leaf tissue was extracted in 3 ml of tri-chloroacetic acid (0.1%) and centrifuged at 4°C at 13, 000 rpm for 10 min. Afterwards, 4 ml of thiobarbituric acid (0.5%) in 20% tri-chloroacetic acid was further added in supernatant. The mixture was placed in a water bath for 30 min at 95°C and immediately cooled by keeping it on an ice bath for reaction termination. Reaction mixture absorbance was monitored at 532 and 600 nm. MDA content was analyzed by taking the difference in absorbance using extinction coefficient, i.e., 155/mm/cm.

### 2.5. Estimation of antioxidant enzymes and ascorbic acid

The enzymatic activity of SOD (EC 1.15.1.1) was evaluated according to the procedure given by Beauchamp and Fridovich (1971). Reaction mixture containing 75 μM NBT, 50 mM potassium phosphate buffer with 2 μM riboflavin, 100 μM EDTA, 13 mM DL-methionine, and 15 μl of enzyme extract was used for estimation of enzymatic activity of SOD. The absorbance was measured at 560 nm. The reaction mixture was illuminated for 30 min at 25°C. Enzymatic activity (1 unit) was revealed as the quantity of enzymes essential for 50% inhibition at 25°C of NBT reduction.

Ascorbic acid was analyzed through the Roe and Kuether (1943) method. 0.1 g of activated charcoal and 4 ml double distilled water was added in the mixture containing 0.5 ml plant extract, and 0.5 ml TCA (50%). The mixture was filtered through Whatman filter paper #1. 0.4 ml of 2, 4-dinitrophenylhydrazine (DNPH) reagent was added to 1 mL of filtrate and incubated for 3 h at 37°C. Afterwards, 1.6 mL of chilled H_2_SO_4_ (65%) was supplemented to mixture and incubated at room temperature for 30 min. The absorbance was recorded at 520 nm.

### 2.6. Statistical analysis

All data is represented as mean value (*n* = 3) ± standard deviation (SD). To study the significance at *p* < 0.05 of the given data, analysis of variance (ANOVA) with LSD *post hoc* tests were done to examine substantial differences through SPSS version 17.0 software.

## 3. Results

**Figure 1:**
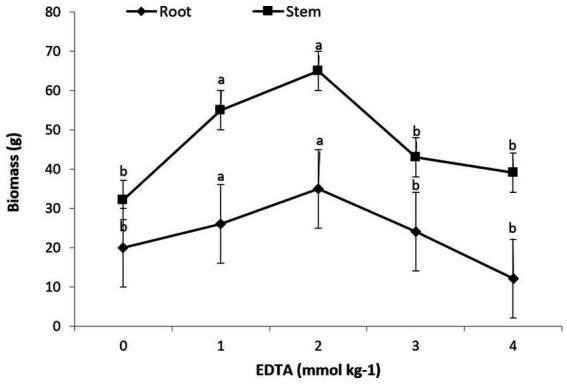
Effect of different EDTA concentration on plant biomass. The vertical bars represent means ± S.D. (*n* = 3). Bars with different letters are significantly different (*p* ≤ 0.05).

A statistical examination of the data revealed that the interaction of heavy metals, EDTA, and Brassica species had a substantial consequence on root as well as shoot biomass. The obtained data indicated that the maximum plant biomass attained for root and shoot was 35 g and 65 g, respectively, with a 2 mM kg^−1^ EDTA application. Furthermore, the maximum concentration of EDTA (3 and 4 mM kg^−1^) given to *B. juncea*’s shoot and root leads to a modest decrease in plant biomass, as depicted in Figure 1. Though there were no signs of rot or chlorosis in the plants, this divulged that *B. juncea* has virtuous tolerance for heavy metals (Pb, Ni, Hg, and Zn,) and EDTA.

### 3.1. Effect of EDTA on heavy metal concentration in soil and plant samples

**Figure 2:**
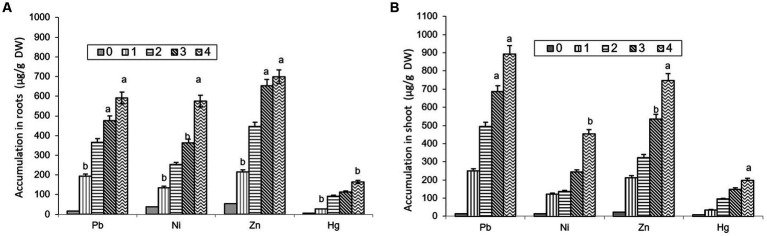
Depiction of heavy metal accumulation in roots **(A)** and shoot **(B)** under different EDTA concentration. The vertical bars represent means ± S.D. (*n* = 3). Bars with different letters are significantly different (*p* ≤ 0.05).

**Figure 3:**
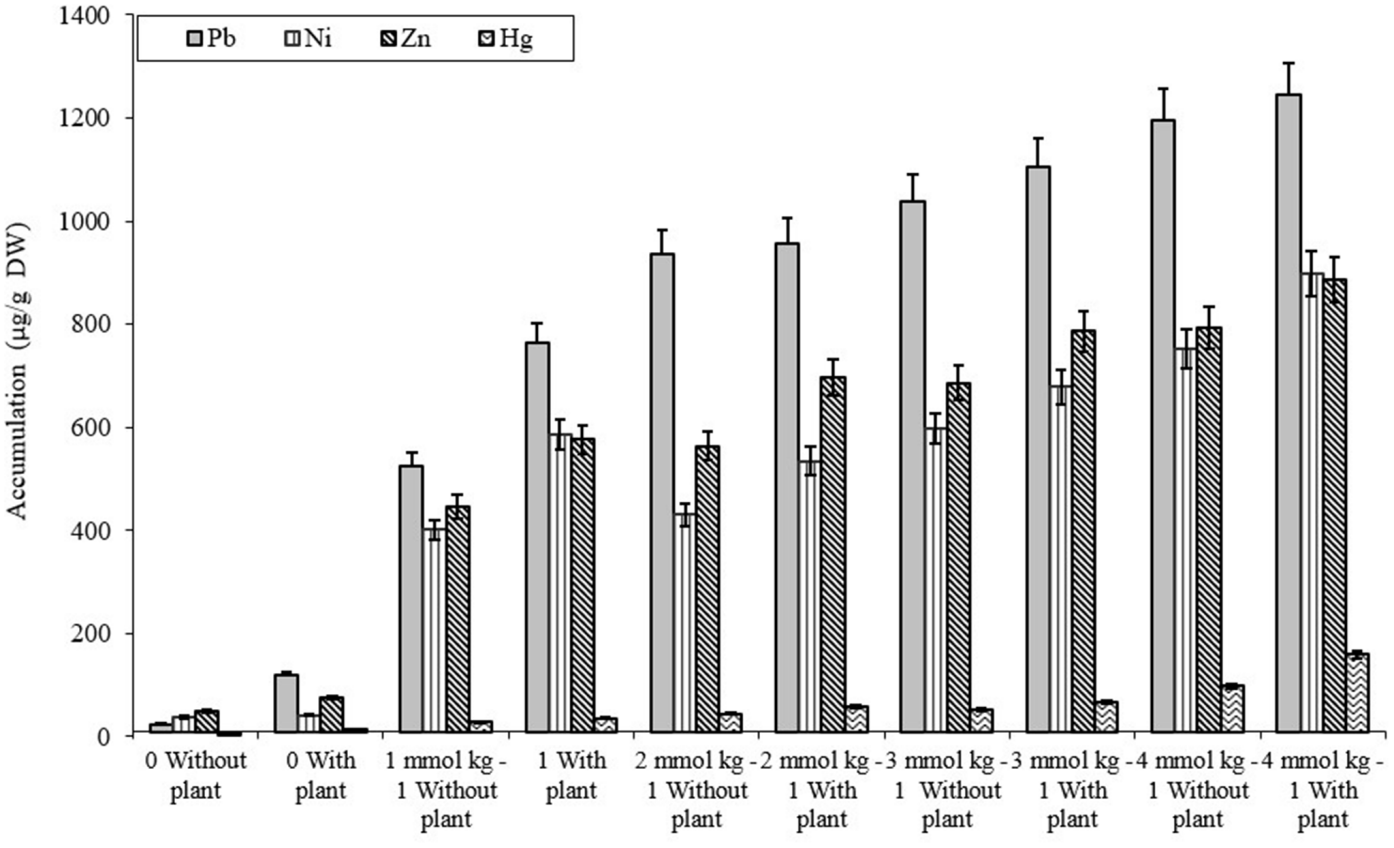
Illustration of heavy metal accumulation in soil under different EDTA concentration. The vertical bars represent means ± S.D. (*n* = 3).

The comparative study of dissimilar concentrations of EDTA divulged that absorption of heavy metals via the roots of *B. juncea* was as follows: Zn > PbNi>Hg, as depicted in Figure 2A, while in shoot Pb > Zn > Ni > Hg (Figure 2B). The phytoremediation effects of water in plants on different elements Pb, Ni, Zn, and Hg were 0.11, 0.33, 0.30, and 0.60 μg/g DW, respectively. It was observed that the effect differs depending on the constituents. The effect of phytoremediation on different elements (Pb, Ni, Zn, and Hg) in the shoot region was changing at different values, but it was identified that at 100, the values were at their maximum and were obtained as 894, 454, 748, and 198 μg/g DW. The procedure was also applied to roots, and the consequences on various elements Pb, Ni, Zn, and Hg were 592, 575, 698, and 165 ug/g DW respectively, but the values were depicted least at a value of 0. The treatment containing high levels of EDTA has encouraged Zn and Pb uptake capacity in the shoot part of *B. juncea.* Heavy metal concentrations in soil have increased by increasing the level of EDTA, as shown in Figure 3. Due to EDTA’s high concentration, plants have absorbed and deposited more heavy metals in the soil.

**Figure 4:**
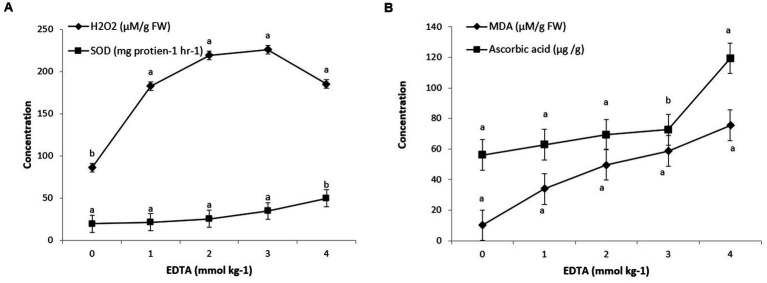
Antioxidant enzyme **(A)** and MDA, ascorbic acid **(B)** activity in *Brassica juncea* seedlings induced by implication of different EDTA concentration under heavy metal stress condition. The vertical bars represent means ± S.D. (*n* = 3). Bars with different letters are significantly different (*p* ≤ 0.05).

The translocation factor (TLF) and transfer factor (TF) in plants were deliberated to predict the heavy metal accretion rate in *B. juncea* under different treatments (Table 1). TF and TLF values in EDTA applications are amplified by enhancing the EDTA levels. The highest TF in Pb was recorded at 1.19 and 1.63 μg/g DW for Zn, with an implication of 4 mM/kg EDTA. Moreover, the highest TF was recorded as 1.26 in the case of Ni with no supplementation of EDTA. Furthermore, a TF value of 3.91 μg/g DW was the maximum in Hg with the application of 3 mM/kg EDTA. While the highest TLF values in Pb and Zn were recorded at 1.51 and 1.07 μg/g DW with supplementation of 4 mM/kg EDTA, In the case of Ni and Hg, the TLF values were recorded as 0.90 and 1.33 μg/g DW in 1 mM/kg EDTA concentration, respectively. On average (EDTA applications only), TLF was found to be in the order Pb > Hg > Zn > and > Ni (Figure 4), while TF was examined in the order Hg > Zn > Ni > Pb.

**Table 1:** Transfer factors and translocation factors for different EDTA concentration.

EDTA Level (mM kg^−1^)	Transfer factor				Translocation factor			
	Pb	Ni	Zn	Hg	Pb	Ni	Zn	Hg
0	0.25	1.26	1.00	1.07	0.76	0.36	0.43	1.29
1	0.58	0.44	0.74	1.75	1.28	0.90	0.99	1.33
2	0.90	0.73	1.10	3.17	1.35	0.54	0.73	1.03
3	1.05	0.90	1.51	3.91	1.44	0.67	0.82	1.32
4	1.19	1.15	1.63	2.28	1.51	0.79	1.07	1.20

### 3.2. Photosynthetic pigment determination

**Figure 5:**
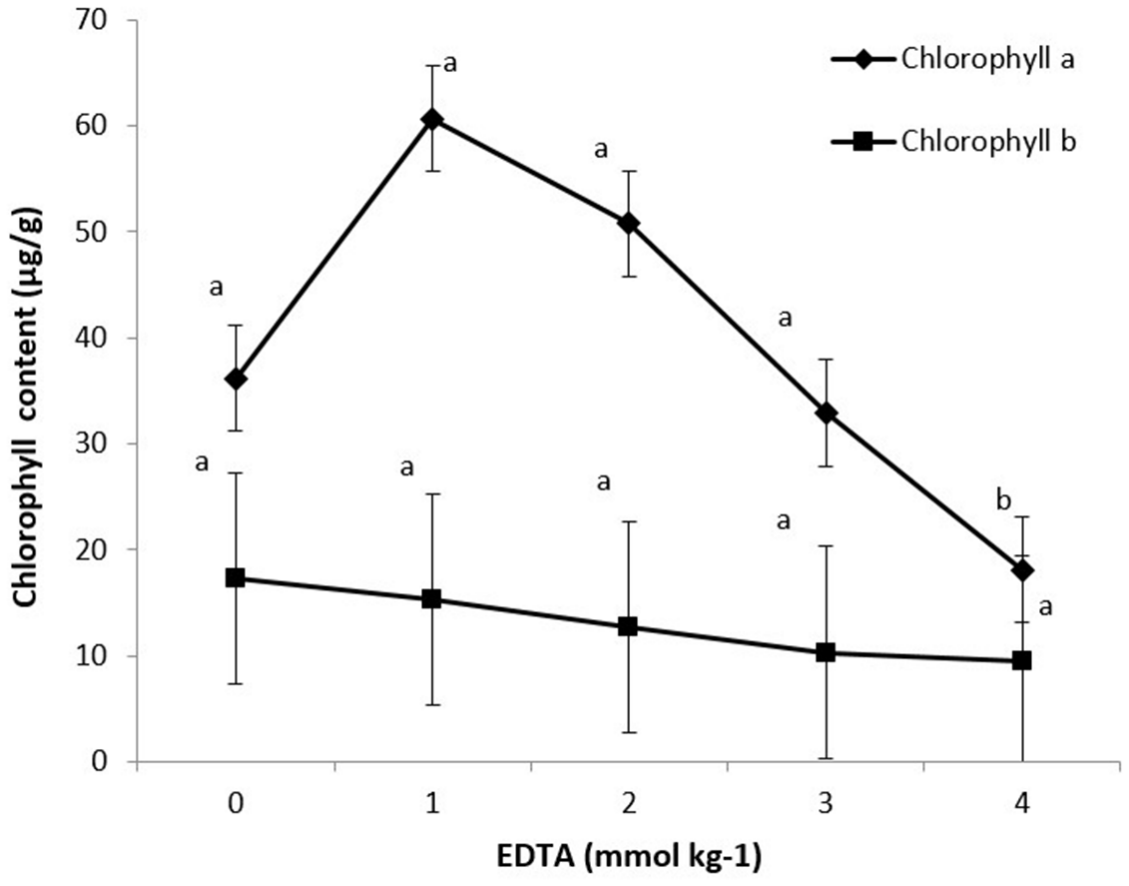
Effect of different EDTA concentration on photosynthetic pigments (Chlorophyll *a* and *b*). The vertical bars represent means ± S.D. (*n* = 3). Bars with different letters are significantly different (*p* ≤ 0.05).

The impact of various EDTA concentrations on photosynthetic pigments (chlorophyll a and b) is shown in Figure 5. Maximum chlorophyll content was recorded at 5.12 in 1 mM EDTA concentration, followed by 3.45 in 0 mM concentration. These data clearly indicate that as EDTA levels increased, heavy metal accumulation subsequently decreased chlorophyll a and b content in plants.

### 3.3. H_2_O_2_ and monoaldehyde (MDA) content activity

*Brassica* sp. seedlings exposed to EDTA revealed that maximum levels of oxidative stress were due to an increase in H_2_O_2_ production. A severe rise in H_2_O_2_ level of 161.64% in 3 mM Kg ^−1^ EDTA-treated seedlings was revealed, contrary to control seedlings (0 mM EDTA). However, a sharp decline in H_2_O_2_ content of 111.58% was examined in seedlings grown with the application of 1 mM Kg^−1^ EDTA. These results clearly signify the effect of EDTA treatments on plant health under oxidative stress. Similarly, *Brassica* seedlings grown with EDTA showed a steep increment in the MDA content (640.19%), contrary to the control (0 mm EDTA). As EDTA concentration increases from 1 to 4 mM Kg^−1^, the lipid peroxidation level in leaves also increases (from 232.35 to 640.19%) (Figure 4).

### 3.4. SOD and ascorbic acid activity

Exogenous EDTA supplementation in heavy metal affected plants raised SOD activity in contrast to plants without EDTA supplementation. As the EDTA concentration increased from 0 to 4 mM/kg, the activity of SOD also increased from 19.6 to 49.7 U min^−1^ mg^−1^ protein. Moreover, non-enzymatic antioxidants like ascorbic acid were correspondingly examined and found to be increased in seedlings under heavy metal stress conditions. A tremendous increment of 11.94% in ascorbic acid content was monitored in seedlings under heavy metal stress with the application of 1 mM Kg^−1^ EDTA, contrary to control, which was further promoted by 23.88, 29.59, and 112.83% with 2, 3, and 4 mM Kg^−1^ EDTA treatment (Figure 4).

## 4. Discussion

HMs uptake through plant rhizosphere and its transportation into the shoots of *B. juncea* plants were enhanced in parallel with stress intensity when HMs was implemented in the medium. HMs mobilization in the rhizosphere, uptake by plant roots, translocation from roots to aerial parts of plants, and heavy metal ion sequestration and compartmentation in plant tissues are some of the steps utilized by plants to extract HMs. Enhanced HMs concentrations result in reductions in elongation, biomass, and seedling growth. A statistical study revealed that HM, EDTA, and the interaction of HM × EDTA with plant species had a substantial effect on both root and shoot length. In the current investigation, heavy metal stress substantially affected the biomass of *B. juncea* seedlings, as observed by a decrease in the fresh and dry weights of the seedlings. Similarly, Qadir et al. (2004) studied *B. juncea* sps. For the efficacy of phytoextraction and found a decrease in shoot length of *B. juncea* subjected to Cd (0.0–2.0 mM). Furthermore, plants imperiled to heavy metal stress have detrimental effects on numerous metabolic cycles because of ROS generation, resulting in reduced plant yield, production, and biomass. Heavy metal toxicity has also been revealed to decrease biomass in *Solanum lycopersicum*, *B. juncea*, *S. seban*, and *S. melongena* (Mahmud et al., 2017; Singh and Prasad, 2019; Din et al., 2020). Zheng et al. (2010) affirmed that application of Cd to the growth medium leads to increased Cd accretion in the root, in contrast to the shoot, which was validated by a greater reduction in root growth compared to shoot growth. Implementing EDTA in the soil decreases free Cd^2+^ ions around the rhizosphere and thereby reduces plant metal uptake. Moreover, Jiang et al. (2003) illustrated that EDTA perhaps decreases the solubility and bioavailability of HMs in the soil system and consequently reduces plant uptake.

Moreover, strong metal chelators like EDTA are known to have a significant impact on chemical speciation, which in turn affects soil solution phase mobility, solubility, and bioavailability as well as root absorption and accumulation of metals. The current study indicated that high-concentration EDTA treatment has encouraged Zn and Pb uptake capability in the shoot part of *B. juncea.* This is confirmed by a previous study that observed the application of EDTA to the soil, which results in heavy metals being phytoextracted and moved from the rhizospheric region to the plant’s harvestable above-ground components (Meers et al., 2008; Singh and Singh, 2017). Several experiments have been done by utilizing soil that was artificially enriched with heavy metals, which may result in high phytoextraction effects (Zhuang et al., 2007) due to the greater accessibility of heavy metals in artificially enriched soils. However, Wu et al. (2004) also concluded that Cu and Pb concentrations were also increased by EDTA application in shoots of *B. juncea*.

The impact of EDTA was examined on the uptake, leaching, and mobilization of heavy metals, along with the effects of EDTA inoculations on *B. juncea.* The most effective EDTA dose was 4 mM EDTA kg^−1^ in soil, where Ni, Pb, and Zn were significantly higher in the shoot biomass, and a 3 mM kg ^−1^ concentration for Hg that was maximum in the shoot biomass, contrary to the control. Implications of EDTA on soil-induced Hg, Ni, Pb, and Zn bioavailability, which consequently stimulated phytoaccumulation and facilitated phytoextraction. Conversely, enhanced Cd and Pb bioavailability also reduced plant growth. The exact mechanism by which EDTA increases metal absorption is still being investigated.

The efficacy of phytoextraction is associated with both plant dry matter generation and heavy metal content. The best plant species for cleaning up a polluted area should be capable of producing the driest matter while tolerating and accumulating the target toxins (Wu et al., 2004; Clemens, 2006; Singh et al., 2017). Heavy metal concentrations by roots and shoot parts are unveiled in Figure 5, depicting less accretion of heavy metals in roots and a relatively high amount of heavy metals translocated to the shoot of *B. juncea.* Moreover, as per Brunetti et al. (2011), who studied *B. napus* plants in polluted soil, the accrual of the several metals under investigation (Cd, Cr, Cu, Ni, Pb, and Zn) was more prominent in shoots than in roots, as is characteristic of accumulator species. Zaier et al. (2010) analyzed the effect of EDTA on *B. napus* to eliminate metals from soils amended with sludge in HM removal, and the study disclosed an improvement in shoot metal accumulation. Furthermore, inoculation with EDTA has also encouraged the accretion of all heavy metals in the roots and shoot parts of *B. juncea*. Grcman et al. (2001) reported high metal (Pb, Zn, and Cd) accumulation performance in *B. rapa*. Moreover, for the disposal of phytoremediation plants with HMs, a variety of techniques including heat treatment, extraction treatment, microbiological treatment, compression landfilling, and nanomaterial synthesis can be applied. Each disposal technique has a unique operation procedure and set of technical requirements. HMs can move and change throughout various disposal procedures. Some techniques of disposal and usage may produce byproducts (Liu and Tran, 2021).

Heavy metal stress is commonly associated with oxidative stress and altered metabolism, such as alteration of chlorophyll biosynthesis, enzymatic activity, and pigment content (Szollosi et al., 2009). Heavy metal-induced oxidative stress also damages chlorophyll. Therefore, chlorosis of leaves is a common deleterious outcome of heavy metal stress. Plants have evolved multiple adaptation mechanisms to protect themselves from heavy metal stress. Disproportionate generation of ROS has deleterious effects on various cellular components, which affect cellular integrity and result in cell death (Askari et al., 2021). Heavy metal stress has damaging consequences for plant biomass and augments ROS production, which hinders the plant’s function (Qureshi et al., 2020). Several plant sps. Can be reconnoitered as phytoremediators to diminish the contrary effects of heavy metals via the modulation of enzymatic and non-enzymatic antioxidants. The current study demonstrated the efficacy of *B. juncea* in the presence of EDTA in ameliorating heavy metal-induced oxidative damage in *B. juncea* seedlings via the production of enzymatic and non-enzymatic antioxidant molecules. The decrease in biomass in seedlings in the presence of heavy metals could be attributed to overproduction of ROS and reduced nutrient and water uptake. Our results are in agreement with Niakan and Kaghazloo (2016) and Farid et al. (2017) who stated that chlorophyll content in *Helianthus annuus* L. and *B. napus* plants increase in the presence of EDTA.

Moreover, SOD enzyme activity in *B. juncea* showed a significant increase with different EDTA concentrations since the increment in EDTA level upsurges the heavy metal concentrations in different plant parts. The enhanced SOD activity was concomitant with reduced O^2• −^ content as the conversion of O^2• −^ to H_2_O_2_ is controlled by SOD. These findings support the earlier work carried out with diverse plant types (Shaw and Mueller, 2009). SODs are a group of enzymes that accelerate the dissociation of superoxide radicals into H_2_O_2_. Several studies have reported the repressive effect of lipid peroxidation in leaf and root tissues under heavy metal stress (Demirbas et al., 2005; Charriau et al., 2016). Malondialdehyde-derived toxic compounds are produced downstream of ROS to mediate metal stress-induced oxidative damage in several crops. Our results clearly signify the consequences of EDTA as well as heavy metals on plant membrane oxidative damage. The non-enzymatic antioxidants, *viz.,* ascorbic acid, are important redox buffering mediators in cells, which regularize oxidative stress by quenching ROS and conserving the redox status of the cell (Noctor et al., 2018). Additionally, ascorbic acid is also engaged in governing numerous plant developmental functions such as cell division and differentiation, homeostasis, pollen growth, phytohormones, etc. (Potter et al., 2012). The findings of the current study showed an elevated level of ascorbic acid in EDTA-treated seedlings under heavy metal stress, which was analogous to the results observed in *O. sativa*, *B. napus,* and *Z. mays* under heavy metal stress (Chen et al., 2017; Ulhassan et al., 2019; Adhikari et al., 2020; Mocek-Płociniak et al., 2023).

## 5. Conclusion

Our study suggests that heavy metal stress significantly reduced the growth, biomass, and photosynthetic pigments of *B. juncea*. It also changes the antioxidative machinery of the plant system due to the overgeneration of ROS and lipid peroxidation. Furthermore, the effects of EDTA on the heavy metal-treated seedlings reduced heavy metal accumulation and upregulated the various nonenzymatic metabolites and antioxidant enzyme activity, which scavenged the toxic ROS (H_2_O_2_ and O^2• −^) from the plant. The most effective EDTA dose was 4 mM EDTA kg − 1 in soil, where Ni, Pb, and Zn were found to be significantly higher in the shoot biomass, and a 3 mM kg −1 concentration for Hg that was maximum in the shoot biomass. Thus, EDTA can be a potent candidate for conferring heavy metal stress, but the underlying molecular mechanism of heavy metal stress should be further elucidated.

## Data availability statement

The original contributions presented in the study are included in the article/supplementary material, further inquiries can be directed to the corresponding author.

## Author contributions

MK, KP, and FK: conceptualization, investigation, and manuscript writing. MK, FK, RS, and OH: data collection and analysis. KP, SB, JS, and MQ: manuscript reviewing and editing. KP, SB, and JS: manuscript finalization and submission. All authors contributed to the article and approved the submitted version.

## Conflict of interest

The authors declare that the research was conducted in the absence of any commercial or financial relationships that could be construed as a potential conflict of interest.

## Publisher’s note

All claims expressed in this article are solely those of the authors and do not necessarily represent those of their affiliated organizations, or those of the publisher, the editors and the reviewers. Any product that may be evaluated in this article, or claim that may be made by its manufacturer, is not guaranteed or endorsed by the publisher.
